# Correlation analysis of the serum CCR5 and LXA4 levels with airway remodelling in bronchial asthma patients

**DOI:** 10.5937/jomb0-61476

**Published:** 2026-06-16

**Authors:** Haiyue Huang, Ruirui Luo, Xuan Yang, Zhigang Wu, Zhongxi Ding

**Affiliations:** 1 Suqian First Hospital, Department of Paediatrics, Suqian City, China; 2 Suqian First Hospital, Department of Nephrology, Suqian City, China; 3 Nanjing University Drum Tower Hospital, Department of Respiratory Medicine, Nanjing City, China; 4 Suqian First Hospital, Department of Emergency Medicine, Suqian City, China

**Keywords:** bronchial, asthma, chemokine receptor 5, lipoxin a4, immune dysfunction, airway remodelling, correlation analysis, bronhijalna astma, receptor za hemokin 5, lipoksin A4, imunoloska disfunkcija, remodelovanje disajnih puteva, analiza korelacije

## Abstract

**Background:**

To analyse the expression levels of serum chemokine receptor 5 (CCR5) and lipoxin A4 (LXA4) in patients with bronchial asthma and their correlations with immune dysfunction and airway remodelling.

**Methods:**

According to disease severity, 240 patients with bronchial asthma admitted to our hospital from January 2023 to May 2025 were divided into mild-to-moderate asthma (n = 180) and severe asthma (n = 60) groups. As the control group, 150 more healthy people who were admitted for a physical examination during that time were chosen. Serum CCR5 and LXA4 levels, as well as immune function indicators (CD 3+, CD 4+, CD8 +, and CD 4+/CD 8+), were measured in each group. The airway remodelling indicators [airway lumen area (LA), airway wall area (WA) and total airway area (TA)] of the right upper lobe tip segment were examined via CT. The correlations between serum CCR5 and LXA4 levels and immune dysfunction, as well as airway remodelling, were analysed using a bivariate Spearman correlation test, and a multivariate logistic model was established to identify independent risk factors influencing serum CCR5 and LXA4 levels in patients with bronchial asthma.

**Results:**

Compared with the mild-to-moderate group, the severe group had higher serum levels of LXA4 and CCR5. Serum CCR5 was higher in the severe group than in the mild to moderate group, and LXA4 was lower in the severe group than in the mild to moderate group. There were statistically significant differences (P&lt;0.05). Serum CD8+ T cell levels were higher than in the control group, whereas CD3+, CD4+, and CD4+/CD8+ T cell levels were lower in the mild to moderate and severe groups. Furthermore, the severe group had lower levels of CD3+, CD4+, and CD4+/CD8+ T cells than the mild-to-moderate group, whereas the mild-to-moderate group had higher levels of CD8+ T cells. The mild-to-moderate and severe groups had lower levels of LA, WA, and TA in the right superior lobe apex segment than the control group, and patients in the severe group had lower levels of these three parameters than those in the mild-to-moderate group. There was a negative connection between serum CCR5 and LXA4 and CD3+, CD4+, and CD4+/CD8+ (P&lt; 0.05) and a positive correlation with CD8+, LA, WA, and TA. Multivariate logistic regression analysis revealed that disease severity, CD3+, CD4+, CD8+, CD 4+/CD 8+, LA, WA, and TA were independent risk factors for increased serum CCR5 and LXA4 in patients with bronchial asthma (P&lt;0.05).

**Conclusions:**

Serum CCR5 and LXA4 are closely related to the severity of bronchial asthma, immune dysfunction and airway remodelling.

## Abstract (sr)

**Uvod:**

Urađeno je ispitivanje nivoa ekspresije serumskog receptora za hemokin 5 (CCR5) i lipoksin A4 (LXA4) kod pacijenata sa bronhijalnom astmom, kao i njihova povezanost sa imunološkom disfunkcijom i remodelovanjem disajnih puteva. Uvod: Urađeno je ispitivanje nivoa ekspresije serumskog receptora za hemokin 5 (CCR5) i lipoksin A4 (LXA4) kod pacijenata sa bronhijalnom astmom, kao i njihova povezanost sa imunološkom disfunkcijom i remodelovanjem disajnih puteva.

**Metode:**

Prema stepenu težine bolesti, 240 pacijenata sa bronhijalnom astmom koji su lečeni u našoj ustanovi od januara 2023. do maja 2025. godine podeljeni su u grupu sa blagom do umerenom astmom (n = 180) i grupu sa teškom astmom (n = 60). Kao kontrolna grupa izabrano je 150 zdravih osoba koje su u istom periodu obavile sistematski pregled. U svim grupama određivani su nivoi serumskog CCR5 i LXA4, kao i pokazatelji imunološke funkcije (CD 3+, CD 4+, CD8 + i CD4+/CD 8+). Pokazatelji remodelovanja disajnih puteva - površina lumena (LA), površina zida (WA) i ukupna površina disajnih puteva (TA) - su procenjeni putem CT snimka segmenta vrha desnog gornjeg režnja. Korelacije između nivoa serumskog CCR5 i LXA4 i parametara imunološke disfunkcije, kao i remodelovanja disajnih puteva, su analizirane Spirmanovim testom korelacije. Za identifikaciju nezavisnih faktora rizika koji utiču na nivoe CCR5 i LXA4 kod pacijenata sa bronhijalnom astmom korišćen je multivarijantni logistički model.

**Rezultati:**

U poređenju sa grupom sa blagom do umerenom astmom, pacijenti sa teškom astmom su imali više serumske nivoe CCR5, a niže nivoe LXA4, pri čemu su razlike bile statistički značajne (P&lt; 0,05). Nivo CD8+ T-celija bio je viši, dok su nivoi CD 3+, CD4+ i CD 4+/CD 8+ T-celija bili niži u grupama sa astmom u odnosu na kontrolnu grupu. U grupi sa teškom astmom su zabeleženi niži nivoi CD 3+, CD4+ i CD 4+/CD 8+, a viši nivoi CD8+ T-celija u poređenju sa grupom sa blagom do umerenom astmom. Vrednosti LA, WA i TA u segmentu vrha desnog gornjeg režnja su bile niže kod pacijenata sa astmom u odnosu na kontrolu, a najniže kod teške forme bolesti. Uočena je negativna korelacija između serumskog CCR5 i LXA4 i pokazatelja CD 3+, CD4+ i CD 4+/CD 8+ (P&lt; 0,05), dok je postojala pozitivna korelacija sa CD 8+, LA, WA i TA. Multivarijantna logistička regresiona analiza pokazala je da su težina bolesti, CD 3+, CD 4+, CD 8+, CD4+/CD8+, LA, WA i TA nezavisni faktori rizika povezani sa povećanim serumskim nivoima CCR5 i LXA4 kod pacijenata sa bronhijalnom astmom (P&lt; 0,05).

**Zaključak:**

Serumski CCR5 i LXA4 pokazali su značajnu povezanost sa težinom bronhijalne astme, imunološkom disfunkcijom i remodelovanjem disajnih puteva.

## Introduction

One form of chronic airway inflammatory illness is bronchial asthma [1][2][3]. Patients frequently experience symptoms such as coughing, wheezing, shortness of breath, and breathing difficulties at the time of disease onset [4]. With the deterioration of the environment, the incidence of bronchial asthma has been increasing annually internationally, and the situation is very serious [5][6][7]. The cause of this disease is related to the mutual influence of various immune cells, including lymphocytes, neutrophils, and airway epithelial cells [8]. The mutual influence of these immune cells can form a complex inflammatory network that, in turn, induces airway hyperresponsiveness. Important pathological features include immune dysfunction and airway remodelling. Moreover, in recent years, studies have increasingly explained the pathogenesis of bronchial asthma in terms of airway immune-inflammatory mechanisms. Therefore, it is imperative to clarify immune dysfunction and airway remodelling in patients with bronchial asthma as early as possible during evaluation of their condition [9]. Chemokines are important regulatory factors of the immune system. By binding to G protein-coupled receptors on the surfaces of inflammatory cells, they can effectively regulate the migration of immune cell subsets and mediate immune processes. Among them, chemokine receptor 5 (CCR5) can regulate the migration and immune function of T cells and monocytes [10][11][12]. Moreover, it can shift immune responses toward different Th cell subtypes and accelerate the progression of inflammation. Serum Lipoxin is a key mediator of the endogenous regulation of anti-inflammatory and pro-regression processes [13]. Among them, serum lipoxin A4 (LXA4) can inhibit inflammatory responses through multiple signalling pathways and promote the resolution of inflammatory symptoms; it is also associated with the emergence and progression of inflammatory disorders [14][15][16].

This study analysed serum CCR5 and LXA4 expression levels in patients with bronchial asthma and their correlations with immune dysfunction and airway remodelling.

## Materials and methods

### General information and grouping

An analysis of 240 bronchial asthma patients who were hospitalised to our hospital from January 2023 to May 2025, including 141 males and 99 females. The ages ranged from 21 to 78 years, with an average of 49.12±4.72 years. The body mass index ranged from 19.5 to 28.0 kg/m^2^, with an average of 24.45±2.52 kg/m^2^. The disease course ranged from 1.5 to 15.0 years, with an average of 7.76±1.12 years. According to the classification of disease severity in the »International Bronchial Asthma Prevention and Treatment Guidelines (2022 Edition)«, 180 patients were assigned to the mild-to-moderate group, and 60 to the severe group. A control group of 150 healthy people, 78 men and 72 women, was selected to undergo physical tests at our hospital during the same time period. With an average age of 48.54±4.65 years, the ages varied from 22 to 76 years. Body mass index ranged from 19 to 28 kg/m^2^, with an average of 24.25±2.54 kg/m^2^.

### Inclusion and exclusion criteria

Inclusion criteria: (1) International Guidelines for the Prevention and Treatment of Bronchial Asthma (2022 Edition); (2) Positive bronchial dilation test; (3) The course of the disease was 1 year; (4) Accompanied by symptoms such as coughing and wheezing; (5) Diffuse or pervasive wheezing sounds were heard in both lungs; (6) Body mass index 18 kg/m^2^, with pacing following the principle of voluntary participation.

Exclusion criteria: (1) Concurrently suffered from pulmonary tuberculosis, pulmonary infection, chronic obstructive lung disease, or other illnesses;(2) Had asthma caused by other reasons; (3) Had taken relevant bronchial asthma treatment drugs within 3 months before the visit; (4) Had malignant tumors; (5) Had organic lesions or liver and kidney dysfunction; (6) Unable to tolerate CT examinations.

### Serological examination

All subjects had 4 mL of fasting venous blood drawn on the day of admission, placed in an ethylene-diaminetetraacetic acid anticoagulant tube, and centrifuged (3,000 r/min, centrifugation radius 8 cm, 10 min), after which the supernatant was collected and stored at -80°C. All serum levels of CCR5 and LXA4 were measured by enzyme-linked immunosorbent assay (ELISA), performed in accordance with the manufacturer's instructions (Shanghai Xitang Biotechnology Co., Ltd.).

Standard solution preparation: 0.5 mL of distilled water was added to the standard solution, which was then mixed well to obtain a 20 ng/mL solution. Before use, 10 mL of distilled water was added, and the mixture was stirred until a solution at 20 nmol/mL was obtained. At the same time, 100 μL of a 20 nmol/mL solution was added to the first tube and mixed. A total of 500 μL was transferred to the second tube. The above operation was repeated. Five hundred microlitres were removed from the seventh tube and discarded. The eighth tube was used as the blank control tube.

(1) Two types of ELISA plates, CCR5 and LXA4, with 96 wells, were used. The plate strips were installed. Six wells were set up for standard solutions, blank control wells, sample wells to be tested, and healthy control wells for the two reagent ELISA plates.

(2) 100 μL of the standard or sample to be tested was added to each well, the mixture was mixed thoroughly, the plate was washed, and the above operation was repeated.

(3) Following the addition of 100 μL of the enzyme-labelled antibody working mixture to each well, the plate was incubated for 10 minutes at 37°C before being cleaned.

(4) Each well received 100 μL of substrate working solution, and the combination was left to react for 15 minutes at 37°C in the dark. Then, each well received 100 μL of stop solution. An enzyme-linked immunosorbent assay (ELISA) reader was used to measure absorbance at 450 nm within 30 minutes, and the corresponding concentration was calculated from the standard curve.

### Detection indices

(1) Serum CCR5 and LXA4 levels: The serum CCR5 and LXA4 levels of patients in the control group, mild to moderate group and severe group were detected.

(2) Immune function markers: CytoFLEX flow cytometry was used to measure the amounts of TLN cells (CD3+, CD4+, CD8+, and CD4+/CD8+) (Beckman Coulter, USA).

(3) Airway remodelling indicators: On the day of admission, a Brilliance CT scanner (Philips, USA) was used to conduct a lung CT examination on the patient. In the inspection method, the patient lies supine with both arms raised. The patient is scanned while holding their breath at the end of inhalation. The scan positions are the thorax and diaphragm. After the scan, the images were uploaded to automated scoring software (Apollo Company, USA) for three-dimensional quantitative analysis.

### Statistical methods

The statistical program SPSS 23.0 was used to analyse the data in this study. The mean and standard deviation (x̄±s) are used to express the measurement data. When comparing two groups, the t-test was used; when comparing several groups, the analysis of variance was used. The χ^2^ test was performed to compare groups, and count statistics are presented as cases or percentages (%). A multivariate logistic model was developed to examine contributing factors, and a bivariate Spearman correlation test was used to assess correlations.

## Results

### Comparison of the serum CCR5 and LXA4 levels among the three groups

A comparison of the three groups' serum CCR5 and LXA4 levels revealed statistically significant differences (P<0.05). Both the mild to moderate group and the severe group had higher serum levels of CCR5 and LXA4 than the control group, and the differences were statistically significant (mild to moderate group vs. control group: t=17.349, 8.557; severe group vs. control group: t=30.027, 31.768, P<0.05). The LXA4 level was lower in the severe group than in the mild to moderate group, while the serum CCR5 level was higher in the severe group. The differences were statistically significant (t=6.907, 4.090, P<0.05) (Table 1).

**Table 1:** Comparison of immune function index levels among the three groups (x̄±s).

Group	Number of cases	CD3+(%)	CD4+(%)	CD8+(%)	CD4+/CD8+
Control group	150	58.94±4.26	36.12±3.18	31.21±3.12	1.72±0.26
Mild to moderate	180	53.68±4.21	33.58±3.15	34.44±3.15	1.42±0.14
Severe group	60	50.16±4.12	30.15±3.12	36.82±3.17	1.12±0.11
F value		37.857	26.669	27.113	76.720
P value		<0.001	<0.001	<0.001	<0.001

### Immune function indicator levels in the three groups

There were statistically significant differences in the levels. There were significant differences in immune function markers among the three groups (P<0.05). While CD8+ cells were higher than in the control group, the mild-to-moderate and severe groups had lower levels of CD3+, CD4+, and CD4+/CD8+ cells compared to the control group. These differences were statistically significant (mild to moderate group vs. control group: t=6.490, 4.193, 7.702, 5.378; severe group vs. control group: t=7.861, 7.133, 9.936, 6.766, P<0.05). The levels of CD3+, CD4+, and CD4+/CD8+ in the severe group were all lower than in the mild to moderate group, while CD8+ levels were higher. These differences were statistically significant (t=3.255, 4.227, 8.715, 2.922, P<0.05) (Table 2).

**Table 2:** Comparison of immune function index levels among the three groups (x̄±s).

Group	Number of cases	CD3+(%)	CD4+(%)	CD8+(%	CD4+/CD8+
Control group	150	58.94±4.26	36.12±3.18	31.21±3.12	1.72±0.26
Mild to moderate group	180	53.68±4.21	33.58±3.15	34.44±3.15	1.42±0.14
Severe group	60	50.16±4.12	30.15±3.12	36.82±3.17	1.12±0.11
F value		37.857	26.669	27.113	76.720
P value		<0.001	<0.001	<0.001	<0.001

### Comparison of airway remodelling index levels among the three groups

The comparison of airway remodelling index levels among the three groups revealed statistically significant differences (P<0.05).

Both the mild to moderate group and the severe group had considerably decreased levels of LA, WA, and TA in the right upper lobe apex segment compared to the control group (mild to moderate group vs. control group: t=6.777, 6.668, 4.919; severe group vs. control group: t=7.149, 7.758, 6.313; P<0.05). Moreover, the levels of LA, WA and TA in the right upper lobe apex segment of the severe group were significantly lower than those in the mild to moderate group (t = 2.510, 3.033, 2.980; P<0.05) (Table 3).

**Table 3:** Comparison of airway remodelling index levels among the three groups (mm^2^, x̄±s).

Group	Number of cases	LA	WA	TA
Control group	150	11.85±1.48	19.87±2.17	29.87±2.48
Mild to moderate group	180	10.04±1.32	17.12±2.14	27.62±2.31
Severe group	60	9.21±1.15	15.45±2.11	25.87±2.16
F value		36.594	38.016	24.160
P value		<0.001	<0.001	<0.001

### Correlations between serum CCR5 and LXA4 levels and immune function and airway remodelling indicators

CD3 + , CD4+, and CD4+/CD8+ showed negative correlations with serum CCR5 and LXA4 (P<0.05), whereas CD8 + , LA, WA, and TA showed positive correlations (P<0.05) (Table 4).

**Table 4:** Correlation between serum CCR5, LXA4 and immune function, airway remodelling indicators.

Indicator	CD3+		CD4+		CD8+		CD4+/CD8+		LA		WA		TA	
	r value	P value	r value	P value	r value	P value	r value	P value	r value	P value	r value	P value	r value	P value
CCR5	-0.814	<0.05	-0.834	<0.05	0.784	<0.05	-0.846	<0.05	0.798	<0.05	0.824	<0.05	0.814	<0.05
LXA4	-0.822	<0.05	-0.831	<0.05	0.793	<0.05	-0.813	<0.05	0.793	<0.05	0.799	<0.05	0.817	<0.05

### Regression analysis of immune dysfunction and airway remodelling

Multivariate logistic regression was conducted with serum CCR5 and LXA4 levels as the dependent variables and age, sex, disease duration, disease severity, CD3+, CD4+, CD8+, CD4+/CD8+, LA, WA, and TA levels as independent variables. In patients with bronchial asthma, elevated serum CCR5 and LXA4 were found to be independently associated with disease severity, CD3+, CD4 + , CD8 + , CD4+/CD8+, LA, WA, and TA (P<0.05) (Table 5).

**Table 5:** Multivariate logistic analysis.

Variable	value	SE (α) value W	aldx^2^ value	OR value	95%CI	P value
CD3+	1.542	0.421	4.845	0.854	0.126 0.964	<0.05
CD4+	1.264	0.463	4.695	0.864	0.128 0.934	<0.05
CD8+	1.165	0.428	4.362	1.795	1.125 1.987	<0.05
CD4+/CD8+	1.254	0.418	4.215	0.852	0.115~0.954	<0.05
LA	1.541	0.436	4.751	1.821	1.117~1.935	<0.05
WA	1.361	0.438	4.361	1.831	1.147~1.934	<0.05
TA	1.221	0.415	4.251	1.824	1.121~1.937	<0.05

## Discussion

The pathogenesis of bronchial asthma is complex and not yet fully elucidated. However, immune dysfunction imbalance is widely regarded as an important basis for the onset of bronchial asthma [17]. The main clinical features are airway hyperresponsiveness and airway remodelling. Airway epithelial cells, various inflammatory cells and their cellular components jointly affect the migration of specific inflammatory cell subpopulations, thereby inducing a series of pathological reactions [18][19][20]. Chemokines and their receptors, as well as numerous inflammatory cells, play increasingly significant roles in the pathogenesis of asthma. In addition, the classical pathological theory holds that an imbalance of Th1/Th2 cells is an important basis for the onset of asthma [21][22][23][24]. LXA4 is expressed in the peripheral blood, alveolar macrophages and neutrophils of adult asthma patients [25]. It is an important anti-inflammatory mediator that regulates the resolution of inflammation. Currently, many studies [26][27][28] have shown that Patients with bronchial asthma have detectable levels of LXA4 in their alveolar lavage fluid, and those who do not receive medication have far higher levels.

In recent years, international studies [29][30][31][32] have confirmed that CCR5 and LXA4 play significant roles in the pathogenesis and clinical manifestations of cerebrovascular disease, allergic purpura, and other diseases. Relevant studies have also revealed that the condition of patients with allergic purpura worsens with increasing serum expression levels. Another study reported that serum CCR5 levels are closely associated with the severity of acute cerebral infarction. However, the above studies did not analyse the impact of serum CCR5 and LXA4 on asthma. In this study, after serum CCR5 and LXA4 levels were measured in healthy individuals undergoing physical examinations and in patients with bronchial asthma of varying severity, patients with mild, moderate, or severe bronchial asthma had higher serum levels of CCR5 and LXA4 [33]. While LXA4 levels were lower in severe patients than in mild-to-moderate patients, serum CCR5 levels were higher in severe patients. It is speculated that increased serum CCR5 and LXA4 levels can activate the inflammatory response, leading to a significant release of inflammatory factors that exacerbate the disease's course and worsen the condition of patients with bronchial asthma [34]. In addition, LXA4 can activate natural killer cells by promoting eosinophil death, thereby reducing the body's immune function and preventing the proinflammatory response of type 2 congenital lymph node-like cells by inhibiting the secretion of inflammatory factors. The lower LXA4 level in the severe study group may be due to highly activated natural killer cells and extensive activation of congenital lymph node-like cells in the heart [35]. Compared with those in the control and mild-to-moderate groups, the levels of serum CD3+, CD4+, and CD4+/CD8+ T cells in the severe group were lower, whereas the level of CD8+ T cells was higher. Multivariate logistic regression analysis also revealed that immune function indicators were influencing factors associated with increases in serum CCR5 and LXA4 levels. Elevated serum CCR5 and LXA4 levels can aggravate immune dysfunction in patients with bronchial asthma. T-cell imbalance impairs immune function, disrupts normal immune cell function, intensifies airway inflammatory responses, aggravates asthma, and ultimately induces pathological reactions such as airway hyperresponsiveness and reversible airflow limitation. CCR5 is expressed in T cells and can regulate T cell proliferation and migration, promote T cell differentiation toward Th17 cells, and thereby mediate inflammatory responses.

LXA4 negatively regulates the body's cellular immune system and interacts with various immune cells and immune regulatory mediators. To a large extent, it determines the direction of the immune response. The upregulation of CCR5 and LXA4 can lead to an imbalance in T cells, disrupt normal immune function, and thereby intensify airway inflammatory responses, thereby aggravating asthma [36]. Moreover, the bivariate Spearman correlation test and multivariate logistic regression analysis both indicated that LA, WA, and TA were associated with serum CCR5 and LXA4 to some extent. It is an independent risk factor affecting the serum CCR5 and LXA4 levels. These findings suggest that elevated serum CCR5 and LXA4 levels can aggravate airway remodelling in patients with bronchial asthma. Airway remodelling is the main pathological change in bronchial asthma and is driven by a vicious cycle of inflammatory injury-repair and reinjury of the airway epithelium. The occurrence and development of airway remodelling are closely associated with changes in levels of inflammatory factors in the airway [37]. The upregulation of serum CCR5 and LXA4 can intensify the aggregation and adhesion of inflammatory cells, thereby increasing the body's inflammatory response and stimulating airway epithelial cells, leading to pathological changes in these cells. This, in turn, aggravates airway hyperresponsiveness and triggers airway remodelling.

## Conclusion

Serum CCR5 and LXA4 are involved in the development and course of bronchial asthma. The severity of a patient's condition, immune function, and airway remodelling can be evaluated by measuring serum levels of CCR5 and LXA4.

## Dodatak

### Institutional review board statement

This study was approved by the Ethics Committee of Medicine (No. HKYS-2025-A0184).

### Conflict of interest statement

All the authors declare that they have no conflict of interest in this work.

## References

[1] Inoue T, Isogai S, Yamamoto N, Hiramatsu N, Niwa Y, Takahashi H, Kimura Y, Horiguchi T, Goto Y, Hashimoto N, Imaizumi K (2024). Safety and efficacy of bronchial thermoplasty in refractory asthma with severe obstructive respiratory dysfunction. Ther Adv Respir Dis.

[2] Savin I A, Zenkova M A, Sen'kova A V (2023). Bronchial Asthma, Airway Remodeling and Lung Fibrosis as Successive Steps of One Process. Int J Mol Sci.

[3] Xu T, Wu Z, Yuan Q, Zhang X, Liu Y, Wu C, Song M, Wu J, Jiang J, Wang Z, Chen Z, Zhang M, Huang M, Ji N (2023). Proline is increased in allergic asthma and promotes airway remodeling. JCI Insight.

[4] Domvri K, Tsiouprou I, Bakakos P, Steiropoulos P, Katsoulis K, Kostikas K, Antoniou K M, Papaioannou A I, Rovina N, Katsaounou P, Papamitsou T, Pastelli N, Tryfon S, Fouka E, Papakosta D, Loukides S, Porpodis K (2025). Effect of mepolizumab in airway remodeling in patients with late-onset severe asthma with an eosinophilic phenotype. J Allergy Clin Immunol.

[5] Yu X, Li L, Cai B, Zhang W, Liu Q, Li N, Shi X, Yu L, Chen R, Qiu C (2024). Single-cell analysis reveals alterations in cellular composition and cell cell communication associated with airway inflammation and remodeling in asthma. Respir Res.

[6] Bajbouj K, Abujabal R, Sahnoon L, Olivenstein R, Mahboub B, Hamid Q (2023). IL-5 receptor expression in lung fibroblasts: Potential role in airway remodeling in asthma. Allergy.

[7] Quan J, Xie D, Li Z, Yu X, Liang Z, Chen Y, Wu L, Huang D, Lin L, Fan L (2024). Luteolin alleviates airway remodeling in asthma by inhibiting the epithelial-mesenchymal transition via β-catenin regulation. Phytomedicine.

[8] Zhang N, Xu J, Jiang C, Lu S (2022). Neuro-Immune Regulation in Inflammation and Airway Remodeling of Allergic Asthma. Front Immunol.

[9] Bessonnat A, Hélie P, Grimes C, Lavoie J P (2022). Airway remodeling in horses with mild and moderate asthma. J Vet Intern Med.

[10] Tiotiu A, Steiropoulos P, Novakova S, Nedeva D, Novakova P, Chong-Neto H, Fogelbach G G, Kowal K (2025). Airway Remodeling in Asthma: Mechanisms, Diagnosis, Treatment, and Future Directions. Arch Bronconeumol.

[11] Huang D, Bai S, Qiu G, Jiang C, Huang M, Wang Y, Zhong M, Fang J, Cheng J, Zhao X, Wu B, Wu D (2024). Myricetin ameliorates airway inflammation and remodeling in asthma by activating Sirt1 to regulate the JNK/Smad3 pathway. Phytomedicine.

[12] Xu L, Huang X, Chen Z, Yang M, Deng J (2024). Eosinophil peroxidase promotes bronchial epithelial cells to secrete asthma-related factors and induces the early stage of airway remodeling. Clin Immunol.

[13] Wu L, Zheng Y, Liu J, Luo R, Wu D, Xu P, Wu D, Li X (2021). Comprehensive evaluation of the efficacy and safety of LPV/r drugs in the treatment of SARS and MERS to provide potential treatment options for COVID-19. Aging (Albany NY).

[14] Wu L, Zhong Y, Wu D, Xu P, Ruan X, Yan J, Liu J, Li X (2022). Immunomodulatory Factor TIM3 of Cytolytic Active Genes Affected the Survival and Prognosis of Lung Adenocarcinoma Patients by Multi-Omics Analysis. Biomedicines.

[15] Alabed M, Elemam N M, Ramakrishnan R K, Sharif-Askari N S, Kashour T, Hamid Q, Halwani R (2022). Therapeutic effect of statins on airway remodeling during asthma. Expert Rev Respir Med.

[16] Ren K, Niu B, Liang H, Xi C, Song M, Chen J, Zhao F, Cao Z (2025). Zhichuanling injection improves bronchial asthma by attenuation airway inflammation and epithelia-mesenchymal transition. J Ethnopharmacol.

[17] Shan L, Chen L, Shen W, Zhou Q, Liu S, Han L, Zhang Q, Dai B, Zhao Y (2024). FOXK2 facilitates the airway remodeling during chronic asthma by promoting glycolysis in a SIRT2-dependent manner. FASEB J.

[18] Wu L, Liu Q, Ruan X, Luan X, Zhong Y, Liu J, Yan J, Li X (2023). Multiple Omics Analysis of the Role of RBM10 Gene Instability in Immune Regulation and Drug Sensitivity in Patients with Lung Adenocarcinoma (LUAD). Biomedicines.

[19] Wang D, Cui Y, Gao F, Zheng W, Li J, Xian Z (2023). Effects of imperatorin on airway remodeling in bronchial asthma through S1PR2/STAT3 signaling pathway. Cell Mol Biol (Noisy-le-grand).

[20] Jesenak M, Durdik P, Oppova D, Franova S, Diamant Z, Golebski K, Banovcin P, Vojtkova J, Novakova E (2023). Dysfunctional mucociliary clearance in asthma and airway remodeling - New insights into an old topic. Respir Med.

[21] Su Z Q, Zhou Z Q, Guan W J, Fan M Y, Rao W Y, Chen Y, Chen X B, Zhong C H, Tang C L, Li S Y, Zhong N S (2022). Airway remodeling and bronchodilator responses in asthma assessed by endobronchial optical coherence tomography. Allergy.

[22] Tsuge M, Ikeda M, Tsukahara H (2022). Novel Lung Growth Strategy with Biological Therapy Targeting Airway Remodeling in Childhood Bronchial Asthma. Children (Basel).

[23] Wu L, Zheng Y, Ruan X, Wu D, Xu P, Liu J, Wu D, Li X (2022). Long-chain noncoding ribonucleic acids affect the survival and prognosis of patients with esophageal adenocarcinoma through the autophagy pathway: construction of a prognostic model. Anticancer Drugs.

[24] Yuan L, Qin Q, Yao Y, Chen L, Liu H, Du X, Ji M, Wu X, Wang W, Qin Q, Xiang Y, Qing B, Qu X, Yang M, Qin X, Xia Z, Liu C (2024). Increased expression of cathepsin C in airway epithelia exacerbates airway remodeling in asthma. JCI Insight.

[25] Wu L, Chen X, Zeng Q, Lai Z, Fan Z, Ruan X, Li X, Yan J (2024). NR5A2 gene affects the overall survival of LUAD patients by regulating the activity of CSCs through SNP pathway by OCLR algorithm and immune score. Heliyon.

[26] Sahnoon L, Bajbouj K, Mahboub B, Hamoudi R, Hamid Q (2025). Targeting IL-13 and IL-4 in Asthma: Therapeutic Implications on Airway Remodeling in Severe Asthma. Clin Rev Allergy Immunol.

[27] Wu L, Li H, Liu Y, Fan Z, Xu J, Li N, Qian X, Lin Z, Li X, Yan J (2024). Research progress of 3D-bioprinted functional pancreas and in vitro tumor models. International Journal of Bioprinting.

[28] Wu L, Zhong Y, Yu X, Wu D, Xu P, Lv L, Ruan X, Liu Q, Feng Y, Liu J, Li X (2022). Selective poly adenylation predicts the efficacy of immunotherapy in patients with lung adenocarcinoma by multiple omics research. Anticancer Drugs.

[29] Galvão J G F M, Cavalcante-Silva L H A, de Almeida Lima É, Carvalho D C M, Alves A F, Mascarenhas S R (2022). Ouabain modulates airway remodeling caused by Th2-high asthma in mice. Int Immunopharmacol.

[30] Wu L, Yang L, Qian X, Hu W, Wang S, Yan J (2024). Mannan-Decorated Lipid Calcium Phosphate Nanoparticle Vaccine Increased the Antitumor Immune Response by Modulating the Tumor Microenvironment. J Funct Biomater.

[31] Ghosh S (2023). Computed tomography findings in bronchial asthma: quantification of air trapping and correlation with pulmonary function tests. Acta Radiol.

[32] Wu L, Li X, Qian X, Wang S, Liu J, Yan J (2024). Lipid Nanoparticle (LNP) Delivery Carrier-Assisted Targeted Controlled Release mRNA Vaccines in Tumor Immunity. Vaccines (Basel).

[33] Behluli E, Spahiu L, Ismaili-Jaha V, Neziri B, Temaj G (2022). Correlation between level of vitamin D in serum and value of lung function in children diagnosed with bronchial asthma. Folia Med (Plovdiv).

[34] Wu D, Wang J, Wei Y, Zhang X, Hou Z (2024). Correlation Analysis of Serum 25-Hydroxyvitamin D Levels With Immune Function and Calcium-Phosphate Metabolism in Patients With Bronchial Asthma Treated With Combination Therapy. Physiol Res.

[35] Wu L, Li X, Yan J (2024). Commentary: Machine learning developed an intratumor heterogeneity signature for predicting prognosis and immunotherapy benefits in cholangiocarcinoma. Transl Oncol.

[36] Scaramozzino M U, Festa M, Levi G, Plastina U R, Sapone G (2023). Correlation between gastroesophageal reflux disease lung volumes and exacerbation of bronchial asthma: Italian pilot observational retrospective study GERDAS. Monaldi Arch Chest Dis.

[37] Zhu Y, Huang B, Jiang G (2024). Correlation between changes in serum YKL-40, LXRs, PPM1A, and TGF-β1 levels and airway remodeling and lung function in patients with bronchial asthma. J Asthma.

